# Silicon tackles butachlor toxicity in rice seedlings by regulating anatomical characteristics, ascorbate-glutathione cycle, proline metabolism and levels of nutrients

**DOI:** 10.1038/s41598-020-65124-8

**Published:** 2020-08-21

**Authors:** Durgesh Kumar Tripthi, Rishi Kumar Varma, Swati Singh, Manisha Sachan, Gea Guerriero, Bishwajit Kumar Kushwaha, Shruti Bhardwaj, Naleeni Ramawat, Shivesh Sharma, Vijay Pratap Singh, Sheo Mohan Prasad, Devendra Kumar Chauhan, Nawal Kishore Dubey, Shivendra Sahi

**Affiliations:** 1 Amity Institute of Organic Agriculture (AIOA), Amity University Uttar Pradesh, Noida, 201313 India; 2 Department of Biotechnology, Motilal Nehru National Institute of Technology, Allahabad, 211004 India; 3 D D Pant Interdisciplinary Research Laboratory, Department of Botany, University of Allahabad, Allahabad, 211002 India; 4 Environmental Research and Innovation Department, Luxembourg Institute of Science and Technology, Hautcharage, Luxembourg; 5 Department of Botany, C.M.P. Degree College, A Constituent Post Graduate College of University of Allahabad, Allahabad, 211002 India; 6 Ranjan Plant Physiology and Biochemistry Laboratory, Department of Botany, University of Allahabad, Allahabad, India 211002; 7 Center of Advanced Study in Botany, Banaras Hindu University, Varanasi, 221005 India; 8 University of the Sciences in Philadelphia (USP), Philadelphia, Pennsylvania USA

**Keywords:** Plant physiology, Plant physiology, Plant physiology, Abiotic, Abiotic

## Abstract

Reckless use of herbicides like butachlor (Buta) in the fields represents a serious threat to crop plants, and hence to their productivity. Silicon (Si) is well known for its implication in the alleviation of the effects of abiotic stresses; however, its role in mitigating Buta toxicity is not yet known. Therefore, this study was carried out to explore the role of Si (10 µM) in regulating Buta (4 µM) toxicity in rice seedlings. Buta reduced growth and photosynthesis, altered nitric oxide (NO) level and leaf and root anatomy, inhibited enzyme activities of the ascorbate-glutathione cycle (while transcripts of associated enzymes, increased except *OsMDHAR*), as well as its metabolites (ascorbate and glutathione) and uptake of nutrients (Mg, P, K, S, Ca, Fe, etc. except Na), while addition of Si reversed Buta-induced alterations. Buta stimulated the expression of Si channel and efflux transporter genes- *Lsi1* and *Lsi2* while the addition of Si further greatly induced their expression under Buta toxicity. Buta increased free proline accumulation by inducing the activity of Δ^1^-pyrroline-5-carboxylate synthetase (P5CS) and decreasing proline dehydrogenase (PDH) activity, while Si reversed these effects caused by Buta. Our results suggest that Si-governed mitigation of Buta toxicity is linked with favorable modifications in energy flux parameters of photosynthesis and leaf and root anatomy, up-regulation of Si channel and transporter genes, ascorbate-glutathione cycle and nutrient uptake, and lowering in oxidative stress. We additionally demonstrate that NO might have a crucial role in these responses.

## Introduction

Rice is a major cereal crop of the world. However, the invasion invasion of weeds is one of the major issues in the rice cultivated areas. To control weeds in the rice fields, several herbicides are being used at larger scale. Among the different herbicides, butachlor (hereafter referred to as Buta) has been extensively used in India for the control of weeds^1,2^. Butachlor, a chloroacetanilide herbicide, (N-[butoxymethyl]-2-chloro-2, 6′-diethylacetanilide), is commonly used in rice fields^3^. In water, the half-life of Buta ranges from 1.65–2.48 days while in soil 2.67–5.33 days^4^. Approximately 6.75 ×10^6^ kg Buta are used annually in India^5^. Besides controlling weeds, the extensive use of herbicides may also affect other crop plants and thus may negatively impact their productivity. Herbicides indeed negatively affect seed germination, hormone synthesis, cell division, cell permeability, nutrient uptake and their assimilation, as well as lipid and protein metabolism^1,6–11^. García-Garijoet al.^12^ have observed that imazamox, a herbicide, drastically decreased root nodule biomass in *Phaseolus vulgaris* and *Vicia sativa*, thereby affecting their nitrogen balance. Herbicides including Buta generally inhibit growth of weeds by affecting the biosynthesis of photosynthetic pigments^13,14^ and either electron carriers or enzymes partaking in photosynthesis^15–17^. Due to their negative interactions with metabolic processes, herbicides including Buta have been reported to cause oxidative stress by inducing the accumulation of reactive oxygen species (ROS) that enhanced lipid peroxidation, and thus, disturbed the fluidity of membranes^2,18–20^. In response to herbicide-created oxidative stress, challenged plants respond by differential activation of antioxidant defense system, and sometimes the magnitude of their activation is not synchronized with oxidative stress, consequently leading to declined biomass production. Further, Burns *et al*.^21^ reported that in *Avena fatua* L. glutathione-related proteins are essential for herbicide resistance. Wang *et al*.^22^ have noticed severe negative impact of nicosulfuron herbicide in the ascorbate-glutathione cycle in *Zea mays* L. Herbicides being persistent and toxic in nature pose serious consequences on plant anatomy, physiology and biochemistry and they can impact, via the food chains, human health too. Therefore, investigations on their ecotoxicology and management strategies are currently highly sought-after.

Silicon (Si) is the 8^th^ most abundant element in nature and the 2^nd^ most abundant element in the earth crust after oxygen^23,24^. The application of Si shows many beneficial effects in grasses, among which an increased mechanical strength^25^. Previous studies showed that Si could improve plant growth and yield by promoting photosynthesis, mineral uptake and antioxidant capacities against environmental constraints^26–28^. Besides, Si also increases the resistance against diseases and pathogens, metal toxicity, salinity, drought, etc.^29–33^. Therefore, over the past years; a clear role of Si in plant biology has emerged. Si is a quasi-essential element controlling growth and development of crops: its effects are latent in the absence of (a)biotic stresses. Indeed, its prophylactic properties are visible upon exogenous constraints. However, the role of Si in mitigating herbicide toxicity like Buta is, to the best of our knowledge, still not elucidated.

Therefore, this study was aimed to explore the role of Si in regulating Buta toxicity in rice seedlings by evaluating growth, photosynthesis, leaf and root anatomical characteristics, oxidative stress, the ascorbate-glutathione cycle, proline metabolism and the regulation of nutrients. Our study has important physiological and ecotoxicological implications and paves the way to follow-up studies on the role of Si under herbicide toxicity in important crops.

## Material and methods

### Plant material and growth conditions

Rice (*Oryza sativa* L.var. Narendra) seeds were bought from the market. Rice seedlings were grown in Petri dishes in a growth chamber^23^ (Impact model IIC 129D, New Delhi) at 28 °C with a daylength of 12 h, relative humidity of 60–70% under 300 µmol photons m^−2^ s^−1^ PFD (photon flux density) for 25 days. Half-strength Hoagland solution was provided regularly to the growing seedlings.

### Preparation of Silicon and Buta combination

After 25 days of growth (seedlings having three well developed leaves), uniformly sized seedlings were selected. Thereafter, treatments of Si (10 µM) and butachlor (Buta, 4 µM) alone and in combinations were given. Our previous study showed that 10 µM of silicon stimulates maximum growth of rice seedlings^33^. However, the selection of Buta dose (4 µM) is based on its dose response curve. It significantly reduced growth (in the terms of fresh weight) of rice seedlings and represents LC_18_. Further, it is an environmentally relevant dose. Sodium silicate salt was used as source of silicon while Buta (EC 50%) was purchased from the market. The samples prepared were: control (only half-strength Hoagland solution), Si (10 µM), Buta (4 µM) and Si (10 µM) + Buta (4 µM). In the case of Si and Buta combination, seedlings were pretreated with Si for 24 h. Separate treatments of 60 ml each were carried out in plastic pots. Five homogeneously-sized rice seedlings were placed in each plastic pot. Then these plastic pots containing the rice seedlings were again kept in the growth chamber for 7 days. Finally, seedlings were harvested from each pot and used for the analysis of various parameters. The experiment was performed in triplicates.

### Determination of growth, protein, photosynthetic pigments, and chlorophyll a fluorescence

Length, fresh and dry weights of both shoot and root were measured as explained in Tripathi *et al*.^33^. Fresh leaves (25 mg) from each sample were extracted in 5 ml of 80% (v/v) acetone for measurement of total chlorophyll and carotenoids content. Lichtenthaler^34^ protocol was used to calculate total chlorophyll and carotenoids in each sample. The amount of protein in each sample was estimated as per the protocol given by Bradford^35^.

FluorPen FP 100, Photon System Instruments, Czech Republic was used to study chlorophyll *a* fluorescence transient in dark-adapted leaves. The following parameters of PS II photochemistry were calculated as per the given formulae of Strasser *et al*.^36^. Subsequently different parameters like yields or flux ratios-Phi_P_0_ (maximum quantum yield of primary photochemistry equivalent to the F_v_/F_m_), Phi_E_0_ (quantum yield of electron transport) and Psi_0 (probability that a trapped exciton moves an electron into the electron transport chain beyond Q_A_), specific energy fluxes per reaction center (RC) i.e. ABS/RC, electron transport flux/RC, TR_0_/RC, trapped energy flux per RC, absorbance flux per RC ET_0_/RC, and DI_0_/RC, dissipated energy flux per RC and performance index (PI_ABS_) were determined.

### Measurement of anatomical parameters

Anatomical changes in root and leaf were studied as described in detail in Tripathi *et al*.^33^.

### Estimation of oxidative markers and lipid peroxidation

The amount of NO_2_¯ formed from hydroxylamine in the presence of superoxide radical (SOR; O_2_^•^¯) was used for measurement of O_2_^•^¯ in each sample as explained by Elstner and Heupel^37^. The absorbance of colored aqueous solution was recorded at 530 nm. Further, the amount of superoxide radicals was determined by the standard curve prepared with sodium nitrite. The quantity of hydrogen peroxide (H_2_O_2_) was measured by the protocol given by Velikova *et al*.^38^. Similarly the standard curve made with a solution of H_2_O_2_ was used to estimate the amount of peroxide radicals in each sample. The lipid peroxidation (LPO) malondialdehyde (MDA) content was determined as explained by Heath and Packer^39^.

### Histochemical and cell viability analyses

*In vivo* localizations of O_2_ – radical, H_2_O_2_, LPO and membrane disruption (MD) was carried out by nitro blue tetrazolium (NBT), 3,3′-diaminobenzidine (DAB), Schiff’s reagent and Evans blue respectively as given by Castro-Mercado *et al*.^40^, Thordal-Christensen *et al*.^41^, Pompella *et al*.^42^ and Schutzendübel *et al*.^43^. Stained leaves were photographed with a Nikon camera, while stained roots were photographed with an Olympus compound dark-field microscope using a digital camera.

Nitric oxide (NO) was detected using the fluorescent dye 4,5-diaminofluorescein diacetate (DAF-2DA), as explained by Xie *et al*.^44^ using a fluorescence microscope (Olympus BX51, Japan).

Pozarowski and Darzynkiewicz^45^ method was used for preparation of plant samples for the cell viability analysis. Briefly leaves were homogenized in phosphate buffer saline (PBS) and centrifuged. 0.5 mL of phosphate buffer saline was added to the pellet so as to completely dissolve the pellet containing 10^6^cells. After fixing in 70% ethanol these cells were kept for incubation at 4 °C for 30 min. Further, cells were washed with 1X phosphate buffer saline and kept for centrifugation at 1200 rpm for 15 min. Supernatant was discarded and the pellet was re-suspended in 200 mL PBS, stained and observed as per the method described in Vishwakarma *et al*.^46^.

### Measurement of enzymes of the ascorbate-glutathione cycle

Enzyme extract for ascorbate peroxidase (APX) glutathione reductase (GR) monodehydroascorbate reductase (MDHAR) and dehydroascorbate reductase (DHAR) were prepared as described in Singh *et al*.^47^. The amount of these enzymes was calculated using UV-visible spectrophotometer (Systronics Model 119, India) at room temperature.

The procedure described by Nakano and Asada^48,49^ was used to quantify APX activity in each sample. One unit (U) of enzyme activity is defined as 1 nmol ascorbate oxidized min^−1^. Schaedle and Bassham^4^ gave the protocol to determine GR activity in different treatments. One unit (U) of enzyme activity is defined as 1 nmol NADPH oxidized min^−1^. MDHAR and DHAR activity was measured as per the method of Hossain *et al*.^50^ and Nakano and Asada^48^ respectively. Subsequently, one unit (U) of enzyme activity for MDHAR is defined as 1 nmol NADPH oxidized min^−1^. One unit (U) of enzyme activity for DHAR is defined as 1 nmol DHA reduced min^−1^. The detailed procedures for these enzymes are given in Singh *et al*.^47^.

### Determination of reduced ascorbate (AsA), dehydroascorbate (DHA), reduced glutathione (GSH) and oxidized glutathione (GSSG)

AsA, DHA, GSH and GSSG were measured as per the procedures of Gossett *et al*.^51^ and Brehe and Burch^52^, respectively, given in detail by Singh *et al*.^47^.

### Gene expression analyses

Liquid nitrogen was used to grind frozen plant tissue (approx. 100 mg) samples in a pre chilled mortar with a pestle. RNA was extracted from the plant tissues according to the procedure given by Spectrum^TM^ plant total RNA Isolation kit (Sigma Life Sciences). The RNA concentration and purity were measured with the Nano drop and the ratio of 260/280 (1.8–2) was maintained. 1 μg of total RNA was used for the synthesis of cDNA using Quanti Tect Reverse Transcription Kit (QIAGEN). PCR conditions and primer details for quantitative reverse transcriptase polymerase chain reaction (qRT-PCR) are given in Table S1. The synthesized cDNA was stored at −20 °C. The gene expression analysis was performed with the Step One Plus Real-Time PCR System (Applied Biosystems), using the SYBR green dye. The reference gene used for analysis was β-actin (housekeeping gene). The result analysis for relative gene expression levels was performed by the standard 2^−ΔΔCT^ method of Livak and Schmittgen^53^. All treatments were analysed in triplicates for the housekeeping gene and all the genes of interest.

### Measurement of proline & its metabolising enzymes

Free proline amount in leaves and roots was estimated as per the method of Bates *et al*.^54^. Toluene was taken as blank and the absorbance of the samples was measured at 520 nm. A standard curve was plotted to determine the amount of proline in each treatment. The activities of proline dehydrogenase (PDH) and Δ^1^-pyrroline-5-carboxylate synthetase (P5CS) in rice seedlings were measured as NADH formation and NADPH oxidation according to the methods of Reno and Splittstoesser^55^ and Garcia-Rios *et al*.^56^, respectively. The procedures are described in detail in Singh *et al*.^57^.

### Estimation of mineral contents

Determination of macronutrients (Mg, P, K,S,Na and Ca) and micronutrient (Fe), and Si in control and treated seedlings was carried out essentially as previously described in Tripathi *et al*.^33^ using an atomic absorption spectrometer.

### Statistical analysis

Duncan’s multiple range test (DMRT) was used to measure the significance of differences between the control and the treatment mean values at *p* < 0.05 significance level. The values presented are the means of three independent experiments (n = 3).

## Results

### Si improves growth, protein and pigments under Buta toxicity

The growth of rice seedlings was significantly decreased by Buta. The decline in growth was 19% in shoot and 26% in root fresh weight, respectively as compared to the control. Similarly, shoot and root dry weight and length were also decreased by Buta. Inhibition in root growth was more under Buta treatment than other growth parameters. On the other hand, supplementation of Si ameliorated Buta-caused reduced growth, as the fresh weight of the shoot and root was comparable to the control (Table 1).

**Table 1:** Effect of silicon (Si) supplementation on growth (root and shoot fresh and dry weight and lengths), protein and photosynthetic pigments of rice seedlings exposed to butachlor (Buta). Data are means ± standard error of three replicates. Values within the same row followed by the different letters are different at *p* < 0.05 according to the Duncan’s multiple range tests.

Parameter	Control	Si	Buta	Si + Buta
Shoot Fresh Weight (g plant^−1^)	0.172 ± 0.002b	0.206 ± 0.003a	0.140 ± 0.002d	0.176 ± 0.003c
Root Fresh Weight (g plant^−1^)	0.082 ± 0.001b	0.098 ± 0.002a	0.061 ± 0.001d	0.081 ± 0.002c
Shoot Dry Weight (g plant^−1^)	0.027 ± 0.0003a	0.028 ± 0.0006a	0.022 ± 0.0003b	0.028 ± 0.0006a
Root Dry Weight (g plant^−1^)	0.009 ± 0.0003a	0.010 ± 0.0001a	0.007 ± 0.0001a	0.008 ± 0.0001a
Shoot Length (cm plant^−1^)	16.2 ± 0.19b	18.7 ± 0.31a	14.1 ± 0.25c	15.7 ± 0.31b
Root Length (cm plant^−1^)	5.0 ± 0.06b	5.9 ± 0.10a	4.0 ± 0.06c	5.2 ± 0.11b
Shoot Protein (mg g^−1^ FW)	15.6 ± 0.19b	16.1 ± 0.27a	12.3 ± 0.22d	14.5 ± 0.28c
Root Protein (mg g^−1^ FW)	10.4 ± 0.13a	10.9 ± 0.18a	9.1 ± 0.16c	9.8 ± 0.19ab
Total chlorophyll (mg g^−1^ FW)	1.74 ± 0.02a	2.04 ± 0.034a	1.38 ± 0.021b	1.71 ± 0.033a
Carotenoids (mg g^−1^ FW)	0.41 ± 0.005b	0.052 ± 0.008a	0.37 ± 0.006c	0.40 ± 0.008b

The protein content in the shoots and roots also decreased by 21 and 12%, respectively. However, Si supplementation alleviated Buta-induced decrease in protein content in both the shoots and roots which showed a reduction of only 7 and 11%, respectively (Table 1).

Similarly to growth and protein content, photosynthetic pigments were also decreased by Buta. The results reveal that Buta decreased total chlorophylls by 21% and carotenoids by 8%. In contrast to this, Si supplementation alleviated the decline in pigment contents and showed a reduction of only 2% in total chlorophylls, while the carotenoids content was comparable to the control (Table 1).

### Si improves PSII photochemistry measured as chlorophyll a fluorescence under Buta toxicity

**Figure 1:**
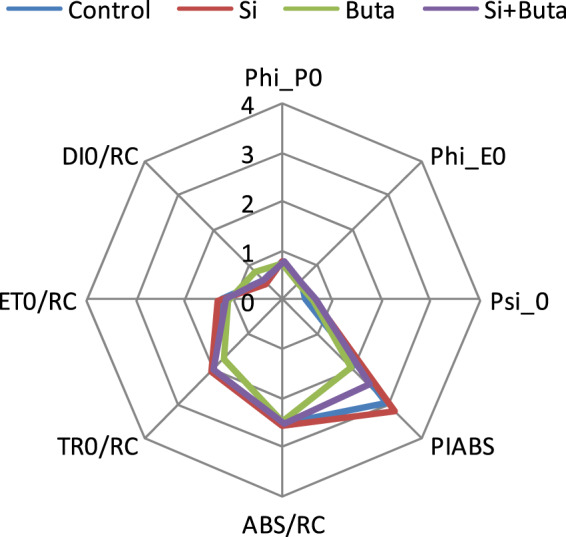
Effect of silicon (Si) supplementation on flux ratios, specific energy fluxes (per reaction centre-RC) and performance index in leaves of rice seedlings treated with butachlor (Buta). Measurements were performed randomly on the second leaf of rice seedlings and repeated three times.

The results show that Phi_P_0_, Phi_E_0_, Psi__0_, ABS/RC, TE_0_/RC and ET_0_/RC were adversely affected by Buta, while under similar treatment DI_0_/RC increased (Fig. 1). The PI_ABS_, that indicates vitality of sample, was also severely affected by Buta. In contrast to this, Si supplementation ameliorated the toxic effect of Buta on PSII photochemistry. These results indicate that Si has a protective impact for PSII photochemistry under Buta toxicity.

### Si causes adaptive changes in leaf and root anatomy under Buta toxicity

**Figure 2:**
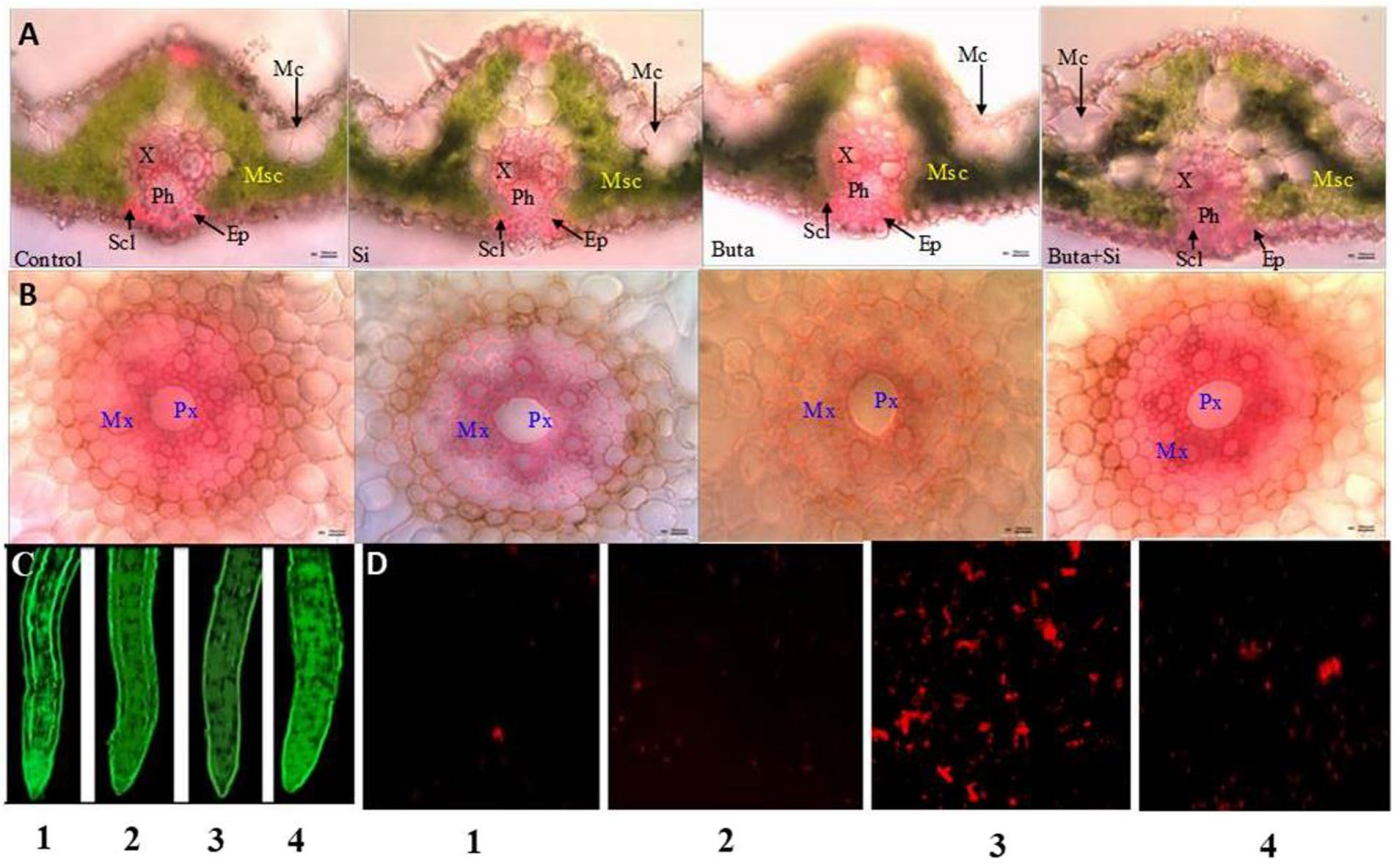
Effect of Si supplementation on leaf and root anatomy of rice seedlings treated with butachlor (Buta). Experiments were repeated three times and gave comparable results. (**A**) Leaf cross sections; (**B**) root cross sections; Msc mesophyll cells; Mc; X xylem; Ph phloem; Pr protoxylem; Mx metaxylem; Scl schlerenchyma; Ep epidermis 0.1 = control; 2 = silicon; 3 = butachlor and 4 = silicon + butachlor. Leaf scale bar 10 µm and root scale bar 50 µm. *In vivo* localizations of (**C**) nitric oxide and (**D**) cell viabilityin rice seedlings treated with butachlor (Buta) and supplemented with silicon (Si). NO was detected with a NO specific fluorescent probe 4,5-diaminofluorescein diacetate (DAF-2DA). Cell viability was measured with the fluorescent probe propidium iodide (PI) staining. 1 =control; 2=silicon; 3=butachlor and 4=silicon+butachlor. Experiments were repeated three times. Scale bar =500 µm.

Buta treatment adversely affected leaf and root anatomy in rice seedlings. Rice seedlings treated with Buta showed reduced leaf thickness of midrib and blade, root diameter, endodermis thickness, size of vascular bundle i.e. xylem and phloem cells (Fig. 2A,B; Tables 2 and 3). The decrease in size of root cells including parenchymatous cells of cortex, xylem, phloem and endodermis thickness ultimately resulted in the reduction of root diameter. Besides this, stomatal length and width were also reduced by Buta, while their numbers were statistically unaffected (Table 2). However, Si application together with Buta improved the anatomical characteristics and restored the size of cells in both leaves and roots. Si addition, together with Buta, showed peculiar modifications in leaf and roots, such as increase in the thickness of leaf midrib and leaf blade, root diameter and endodermis width which might have presented barriers against Buta uptake; hence, the amelioration of Buta toxicity by Si addition was an obvious result (Tables 1–3).

**Table 2:** Effect of silicon (Si) on leaf anatomical characteristics of rice seedlings exposed to butachlor (Buta).Data are means ± standard error of three replicates. Values within the same row followed by the different letters are different at *p* < 0.05 according to the Duncan’s multiple range tests. VB vascular bundle.

Parameter	Control	Si	Buta	Si + Buta
Leaf midrib thickness (µm)	110 ± 4c	129 ± 6a	105 ± 2d	118 ± 3b
Leaf blade thickness (µm)	58.9 ± 2c	68.0 ± 2a	56.7 ± 0.4d	64.6 ± 1b
VB height (µm)	46.6 ± 2b	53.1 ± 2a	42.7 ± 0.2c	45.6 ± 1b
VB width (µm)	49.3 ± 0.6b	52.7 ± 2a	46.4 ± 1c	48.8 ± 0.6b
Metaxylem diameter (µm)	15.8 ± 0.2b	16.8 ± 0.6a	14.7 ± 0.6c	15.7 ± 0.2b
Phloem diameter (µm)	15.4 ± 0.6b	17.8 ± 1a	14.6 ± 0.3c	15.1 ± 0.2b
Epidermal length (µm)	10.9 ± 1b	13.8 ± 0.8a	10.6 ± 0.8c	10.4 ± 0.4b
Epidermal width (µm)	8.6 ± 0.3a	8.9 ± 0.6a	7.9 ± 0.5b	8.5 ± 0.6a
Stomatal length (µm)	24.9 ± 2b	28.1 ± 1a	22.1 ± 0.4c	24.5 ± 0.8b
Stomatal width (µm)	2.9 ± 0.3b	3.4 ± 0.3a	2.7 ± 0.1c	2.9 ± 0.3b
Stomatal number	10.0 ± 0.1b	12.0 ± 0.2a	9.0 ± 0.2c	10.0 ± 0.2b

**Table 3:** Effect of silicon (Si) on root anatomical characteristics of rice seedlings exposed to butachlor (Buta).Data are means ± standard error of three replicates. Values within the same row followed by the different letters are different at *p* < 0.05 according to the Duncan’s multiple range test.

Parameter	Control	Si	Buta	Si + Buta
Root diameter (µm)	507 ± 19c	608 ± 4a	446 ± 15d	518 ± 7b
Exodermis thickness (µm)	14.9 ± 0.2b	15.6 ± 0.3a	12.9 ± 1c	14.4 ± 0.5b
Cortex thickness (µm)	179 ± 7c	194 ± 3a	160 ± 15d	184 ± 10b
Endodermis width (µm)	12.2 ± 1b	12.8 ± 0.6a	11.5 ± 0.1c	11.9 ± 0.3b
Central cylinder diameter (µm)	153 ± 8b	169 ± 4a	141 ± 6d	145 ± 3b
Metaxylem diameter (µm)	36.8 ± 0.5b	40.4 ± 1a	32.8 ± 1c	35.2 ± 2b
Protoxylem diameter (µm)	12.8 ± 0.9b	13.9 ± 0.9a	12.2 ± 1c	12.7 ± 0.6b
Phloem diameter (µm)	8.02 ± 0.3b	8.8 ± 0.6a	7.5 ± 0.2c	7.9 ± 0.4b

### Regulation of NO accumulation and cell viability (or cell death) by Si under Buta toxicity

Buta treatment reduced NO accumulation in roots of rice seedlings (Fig. 2C). However, after Si supplementation together with Buta, the accumulation of NO was higher even compared to that of the control, as indicated by the greater NO fluorescence under Si+Buta combination (Fig. 2C). It indicates that NO might have a positive role in Si-mediated amelioration of Buta toxicity. NO accumulation was also stimulated by Si alone.

The results of cell viability or cell death showed that, under Buta treatment, the number of viable cells was lower, while Si addition with Buta increased cell viability (Fig. 2D). These results clearly indicate that Si protects rice seedlings against Buta toxicity.

### Si lowers oxidative stress biomarkers: ROS accumulation nunder Buta toxicity

**Figure 3:**
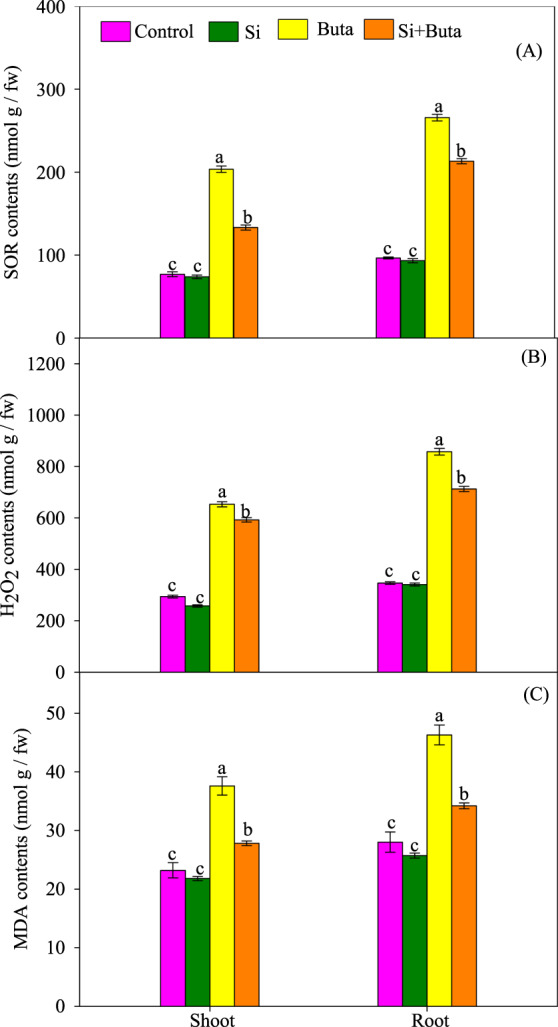
Effect of silicon (Si) supplementation on superoxide radical (O_2_^•–^; a-b), hydrogen peroxide (H_2_O_2_; c-d) and malondialdehyde (MDA; e-f) of rice seedlings treated with butachlor (Buta). Data are means ± standard error of three replicates. Bars with different letters show significant differences at *p* < 0.05 between treatments according to the Duncan’s multiple range test.

Oxidative stress markers were measured in the different conditions analysed: superoxide radical (SOR, O_2_^•−^), hydrogen peroxide (H_2_O_2_) and MDA contents in shoot and root of rice seedlings are presented in Fig. 3. Buta increased (*p* < 0.05) O_2_^•−^ by 53 and 63% in shoots and roots, respectively with respect to the control. Similarly, H_2_O_2_ increased by 52% in shoots and 76% in roots compared to the control. Si supplementation significantly (*p* < 0.05) decreased O_2_^•−^ and H_2_O_2_ contents under Buta toxicity (Fig. 3). Under Buta toxicity, the accumulation of ROS significantly (*p* < 0.05) accelerated lipid peroxidation (MDA formation) as the value increased by 34 and 36% in shoots and roots, respectively, as compared to the control (Fig. 3). In contrast to this, Si supplementation significantly (*p* < 0.05) lowered MDA content as compared to the amount recorded under Buta toxicity alone (Fig. 3).

**Figure 4:**
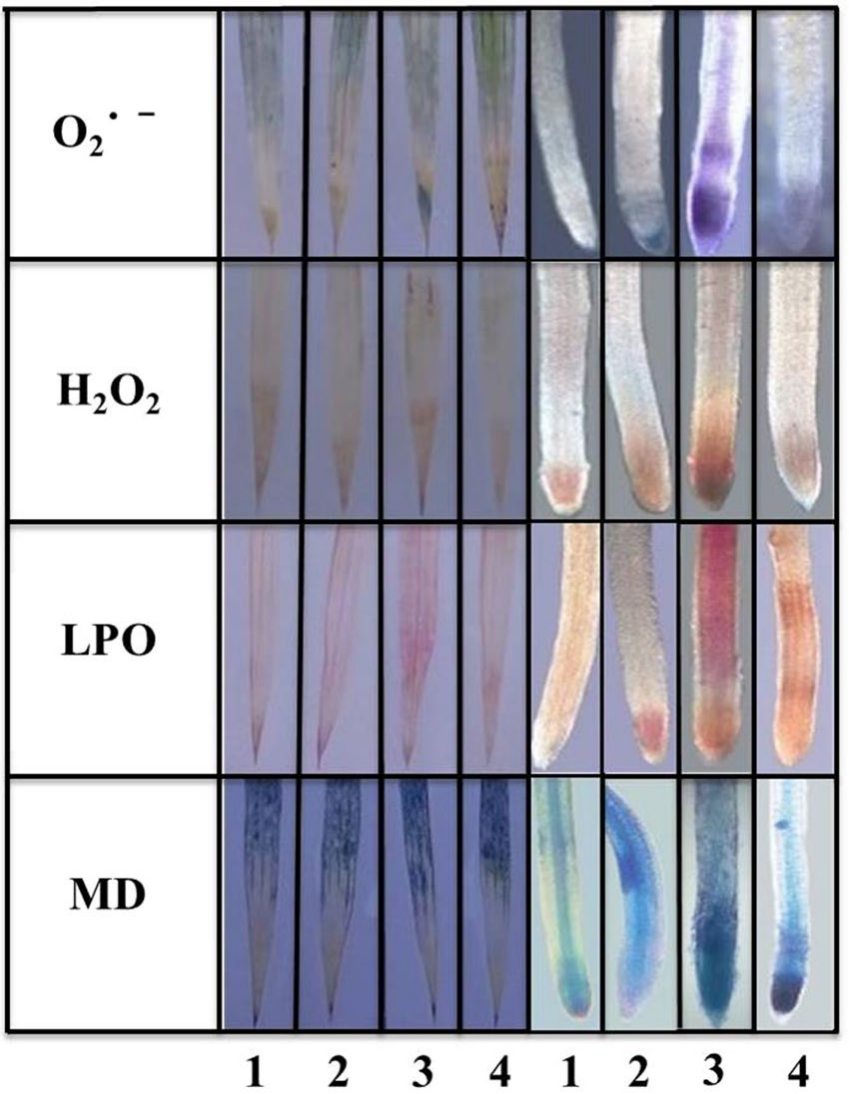
*In vivo* localizations of superoxide radical (O_2_^•–^), hydrogen peroxide (H_2_O_2_), lipid peroxidation (LPO) and membrane damage (MD) in rice seedlings treated with butachlor (Buta) and supplemented with silicon (Si). For *in vivo* localization of O_2_^•–^, H_2_O_2_, LPO and MD nitrobluetetrazolium (NBT), 3,3’-diaminobenzidine (DAB), Schiff’s reagent and Evans blue, respectively were used. 1 =control; 2=silicon; 3=butachlor and 4=silicon+butachlor. Experiments were repeated three times. Scale bar =500 µm.

*In vivo* localizations of O_2_^•–^, H_2_O_2_, lipid peroxidation (LPO) and membrane damage (MD) are shown in Fig. 4. The results showed that Buta accelerated ROS accumulation which resulted in significant amount of LPO and MD in rice seedlings. However, under Si+Buta combination, ROS accumulation, LPO and MD were noticeably decreased. These results indicate that Si addition regulates ROS accumulation, LPO and MD in rice seedlings under Buta toxicity; less oxidative stress, hence better growth, was noticed under Si+Buta combination, as compared to Buta treatment alone.

### Regulation of influx and efflux channel and Si transporter genes of Si by the addition of Si under Buta toxicity

**Figure 5:**
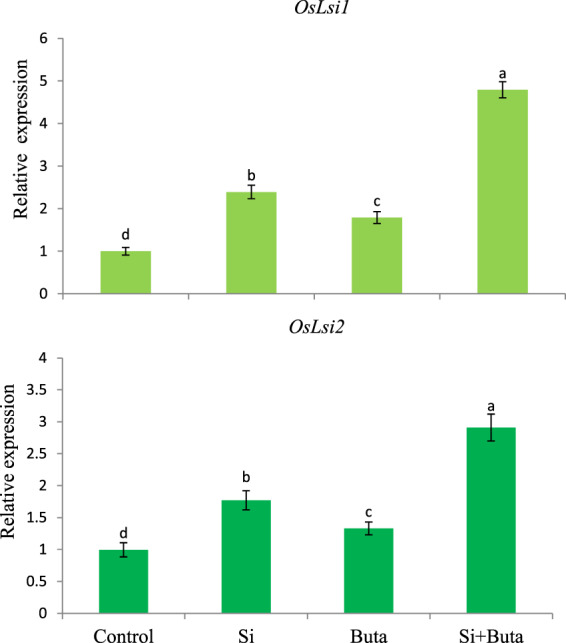
Effect of silicon (Si) supplementation on accumulation of transcripts of Si channel and efflux transporter i.e. *OsLsi1* and *OsLsi2* in roots of rice seedlings treated with butachlor (Buta). Data are means ± standard error of three replicates. Bars with different letters show significant differences at *p* < 0.05 between treatments according to the Duncan’s multiple range test.

The results relative to the expression of the genes encoding the Si channel and transporter genes- *Lsi1* and *Lsi2*, are shown in Fig. 5. The gene coding for the Si channel (*Lsi1*) and efflux transporter (*Lsi2*) though stimulated by Buta (79 and 24%, respectively), were greatly enhanced by the addition of Si under Buta toxicity. Under Si+Buta combination, the relative expression of *Lsi1* and *Lsi2* was enhanced by 379 and 193%, respectively. Si alone also stimulated the expression of *Lsi1* and *Lsi2* (139 and 78%, respectively) and even more than Buta treatment alone (Fig. 5). These results indicate that the enhanced expression of Si channel and transporter genes by the addition of Si is needed in order to restrict Buta entry, as supported by our Si accumulation data.

### Si improves the activity of enzymes of the ascorbate-glutathione cycle under Buta toxicity

The results relative to the enzymes of the ascorbate-glutathione cycle are shown in Table 4. Buta treatment inhibited APX, GR, MDHAR and DHAR activity in shoot and root by 50 and 52%, 38 and 35%, 40 and 40% and 23 and 18%, respectively in comparison to the control. However, Si treatment alone stimulated APX, GR, MDHAR and DHAR activity in shoots and roots by 0 and 8%, 18 and 5%, 10 and 24% and 14 and 22%, respectively as compared to the control. Additionally, when Si was supplemented with Buta, it significantly (*p* < 0.05) further stimulated the activity of APX, GR, MDHAR and DHAR, even in comparison to their respective controls (Table 4).

**Table 4:** Effect of silicon (Si) supplementation on activities of ascorbate peroxidase (APX), glutathione reductase (GR), monodehydroascorbate reductase (MDHAR) and dehydroascorbate reductase (DHAR) in rice seedlings treated with butachlor (Buta). Data are means ± standard error of three replicates.Values within the same row followed by the different letters are different at *p* < 0.05 according to the Duncan’s multiple range test.

Parameter	Control		Si		Buta		Si + Buta	
	Shoot	Root	Shoot	Root	Shoot	Root	Shoot	Root
APX (U/mg protein)	3230 ± 84d	2870 ± 86 f	3424 ± 137b	3099 ± 87e	1647 ± 41 g	1521 ± 61 h	3617 ± 94a	3329 ± 83c
GR (U/mg protein)	187 ± 5c	154 ± 3e	220 ± 6b	162 ± 5d	116 ± 3 f	100 ± 2fg	278 ± 7a	214 ± 6b
MDHAR (U/mg protein)	405 ± 11d	382 ± 11e	445 ± 12c	473 ± 13a	243 ± 6 f	229 ± 6 g	506 ± 14a	477 ± 13b
DHAR(U/mg protein)	489 ± 14d	442 ± 16e	557 ± 17c	539 ± 15c	376 ± 10 f	362 ± 10fg	660 ± 17a	579 ± 18b

### Si regulation of genes coding for ascorbate-glutathione cycle enzymes under Buta toxicity

**Figure 6:**
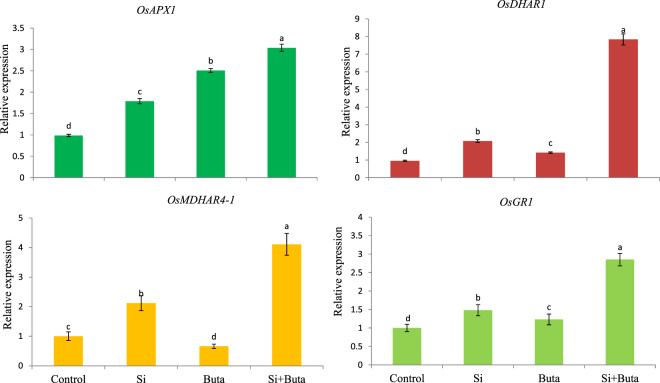
Effect of silicon (Si)supplementation on accumulation of transcripts of (*OsAPX*), glutathione reductase (*OsGR*), monodehydroascorbate reductase (*OsMDHAR*) and dehydroascorbate reductase (*OsDHAR*) in leaves of rice seedlings treated with butachlor (Buta). Data are means ± standard error of three replicates. Bars with different letters show significant differences at *p* < 0.05 between treatments according to the Duncan’s multiple range test.

The results relative to the transcript accumulation of *OsAPX, OsMDHAR, OsDHAR* and *OsGR* are provided in Fig. 6. In contrast to the activities of APX, MDHAR, DHAR and GR, the expression of *OsAPX, OsGR* and *OsDHAR* genes was stimulated by Buta, while the expression of *OsMDHAR* (by 34%) was drastically decreased. However, Si addition together with Buta greatly increased the expression of *OsAPX, OsGR, OsMDHAR* and *OsDHAR* genes by 207, 185, 311 and 719%, respectively (Fig. 6). Si alone also had a stimulatory effect on the expression of these genes (Fig. 6).

### Si maintains the redox status of ascorbate and glutathione: reduced ascorbate (AsA), dehydroascorbate (DHA), reduced glutathione (GSH) and oxidized glutathione (GSSG) under Buta toxicity

The results relating to the redox status of AsA and GSH are shown in Table 5. Buta significantly (*p* < 0.05) reduced the levels of AsA and GSH which results into a decline in ratios of AsA/DHA and GSH/GSSG. Under Buta treatment, ratios of AsA/DHA and GSH/GSSG in shoots and roots were 6.29 and 4.77 and 6.31 and 6.09, respectively. However, the addition of Si with Buta improved AsA and GSH which results into higher ratios of AsA/DHA and GSH/GSSG as they were 9.80 and 7.97 and 11.07 and 9.90 in shoots and roots, respectively. These results indicate that under Buta toxicity, the addition of Si re-establishes the redox status of AsA and GSH and hence protects the cell against oxidative stress.

**Table 5:** Effect of silicon (Si) supplementation on reduced ascorbate (AsA), dehydroascorbate (DHA), AsA/DHA ratio, reduced glutathione (GSH), oxidized glutathione (GSSG) and GSH/GSSG ratio in rice seedlings treated with butachlor (Buta). Data are means ± standard error of three replicates.Values within the same row followed by the different letters are different at *p* < 0.05 according to the Duncan’s multiple range tests. FW = fresh weight.

Parameter	Control		Si		Buta		Si + Buta	
	Shoot	Root	Shoot	Root	Shoot	Root	Shoot	Root
AsA µmol/g FW	1.92 ± 0.0691a	1.55 ± 0.0401c	1.95 ± 0.058a	1.66 ± 0.066b	1.58 ± 0.057b	1.19 ± 0.035d	2.01 ± 0.052a	1.57 ± 0.041b
DHA µmol/g FW	0.231 ± 0.006b	0.208 ± 0.007c	0.219 ± 0.009c	0.206 ± 0.005c	0.251 ± 0.010a	0.249 ± 0.007a	0.205 ± 0.006c	0.197 ± 0.006c
AsA/DHA	8.31 ± 0.291c	7.45 ± 0.268 f	8.90 ± 0.356b	8.06 ± 0.242d	6.29 ± 0.226d	4.77 ± 0.167d	9.80 ± 0.274a	7.97 ± 0.215e
GSH µmol/g FW	1.03 ± 0.0419c	0.866 ± 0.024e	1.11 ± 0.032b	1.04 ± 0.037d	0.915 ± 0.023d	0.750 ± 0.019 f	1.65 ± 0.038a	1.07 ± 0.031b
GSSG µmol/g FW	0.109 ± 0.003b	0.102 ± 0.003b	0.109 ± 0.002b	0.112 ± 0.003b	0.145 ± 0.004a	0.123 ± 0.003b	0.149 ± 0.004a	0.108 ± 0.003b
GSH/GSSG	9.44 ± 0.274d	8.49 ± 0.220 f	10.18 ± 0.305b	9.28 ± 0.269e	6.31 ± 0.151 g	6.09 ± 0.158 h	11.07 ± 0.288a	9.90 ± 0.277c

### Si regulates proline metabolism under Buta toxicity

**Figure 7:**
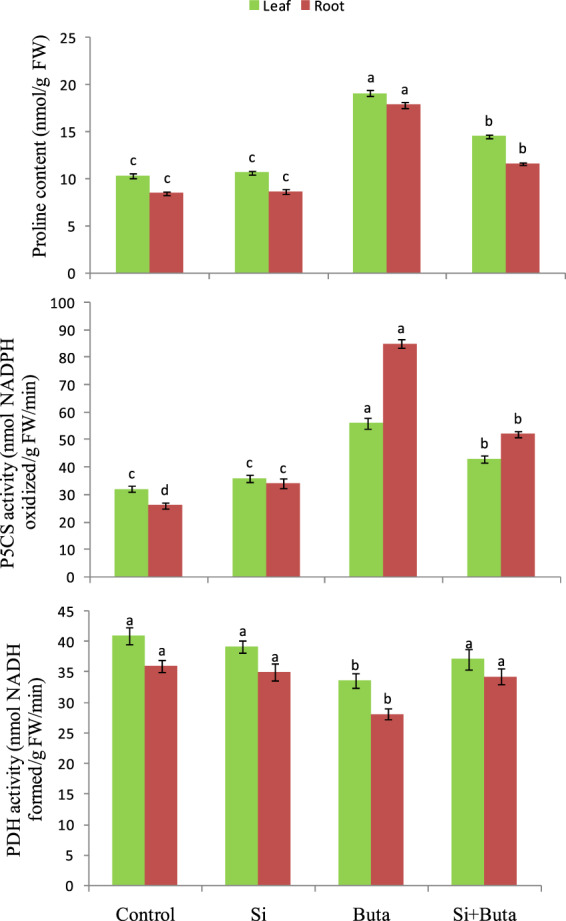
Effect of silicon (Si) supplementation on proline content, and activities of Δ^1^-pyrroline-5-carboxylate synthetase (P5CS) and proline dehydrogenase (PDH) in rice seedlings treated with butachlor (Buta). Data are means ± standard error of three replicates. Bars with different letters show significant differences at *p* < 0.05 between treatments according to the Duncan’s multiple range test.

The results show that in leaves and roots free proline accumulation (85 and 111%, respectively) and activity of P5CS enzyme (by 75 and 227%, respectively) was stimulated by Buta, while Si addition caused lowering in these physiological parameters (Fig. 7). The activity of proline catabolic enzymes i.e. PDH significantly decreased by Buta in leaves and roots (18 and 21%, respectively), while under combination of Si, its activity was comparable to the respective controls (Fig. 7).

### Si regulates accumulation of mineral contents under Buta toxicity

The mineral content of rice seedlings is presented in Table 6. Data relative to Si accumulation indicates that Buta decreased its content in shoots, while its accumulation was stimulated (12%) in roots in comparison to the control (Table 6). However, Si addition together with Buta greatly stimulated its accumulation in shoots and roots. Buta decreased the contents of macronutrients (Mg, P, K, S, and Ca) and micronutrient (Fe) in both shoots and roots and this effect was greater for S and Mg, while Na content increased. However, Si supplementation together with Buta stimulated the accumulation of Mg, P, K, S, Ca and Fe even over the value of control. Si alone also had a stimulatory impact on the accumulation of nutrients. But in the case Na accumulation, Si did not have significant impact. These results indicate that under Buta toxicity, the addition of Si positively regulated the accumulation of the studied nutrients (Table 6).

**Table 6:** Effect of silicon (Si) on mineral contents (mg/kg dry weight) of rice seedlings exposed to butachlor (Buta). Data are means ± standard error of three replicates. Values within the same row followed by the different letters are different at *p* < 0.05 according to the Duncan’s multiple range tests.

Parameter	Control	Si	Buta	Si + Buta
Shoot Si	10958 ± 351c	14902 ± 307a	8985 ± 342d	13588 ± 378b
Root Si	1258 ± 25d	2314 ± 24b	1409 ± 22c	2440 ± 36a
Shoot Mg	4678 ± 117c	5454 ± 142a	3929 ± 156d	4977 ± 178b
Root Mg	3952 ± 158c	4335 ± 171b	3241 ± 185d	4466 ± 168a
Shoot P	8280 ± 167c	9828 ± 145b	7204 ± 165d	10630 ± 325a
Root P	4125 ± 114c	5135 ± 145b	3382 ± 164d	5878 ± 195a
Shoot K	6934 ± 180c	8459 ± 187b	6240 ± 167d	8622 ± 178a
Root K	6725 ± 155c	8403 ± 135b	5716 ± 145c	8775 ± 174a
Shoot S	7603 ± 213c	8591 ± 189b	6006 ± 201d	9275 ± 191a
Root S	7230 ± 156bc	8748 ± 168b	5350 ± 185d	9254 ± 185a
Shoot Na	3578 ± 172b	3506 ± 201b	3936 ± 178a	3665 ± 186b
Root Na	3154 ± 201bc	3058 ± 189c	3512 ± 195a	3257 ± 206b
Shoot Ca	16304 ± 424b	18221 ± 398a	14836 ± 415c	18717 ± 465a
Root Ca	12355 ± 324b	14527 ± 293a	10007 ± 356c	14699 ± 378a
Shoot Fe	284 ± 11c	312 ± 14b	261 ± 14d	353 ± 16a
Root Fe	262 ± 17c	299 ± 16b	223 ± 10d	338 ± 15a

## Discussion

The use of herbicides like Buta has been shown to adversely affect crop growth^3,9–11^. Consistent with these studies, Buta treatment decreased the growth of rice seedlings (Table 1). The decline in growth is linked with Buta-induced alterations in PS II photochemistry, decreased cell viability (Figs. 1 and 3D), and down-regulation of NO accumulation (Fig. 3C) and the ascorbate-glutathione cycle (Tables 4 and 5). Buta triggers the excessive accumulation of ROS, which cumulatively affected anatomy (Tables 2–3, Fig. 2A,B) and physiology of rice seedlings (Table 1). Between shoots and roots, Buta treatment more severely affected root growth parameters (Table 1). Similar to our results, Wang *et al*.^3^ observed more sensitivity in roots than shoots to Buta in Italian ryegrass. Although the Si-mediated alleviation of abiotic stress is well studied^28–32^, its role in the regulation of herbicide toxicity like Buta is not yet known. We noticed that supplementation of Si decreased the level of ROS and cell death (Figs. 3, 4 and 2D), up-regulated the ascorbate-glutathione cycle, as well as the expression of their associated genes (Tables 4 and 5), thereby favoring adaptive changes in anatomical characteristics (Fig. 2A,B; Tables 2 and 3) which resulted in a better growth of rice seedlings under Buta toxicity. Further, the results showed that Si addition together with Buta greatly increased Si accumulation (Table 6) and the expression of the Si channel and transporter genes, *Lsi1* and *Lsi2*, in roots (Fig. 5). These data can be considered as an adaptive strategy resulting in the formation of a barrier to Buta uptake as reported in the case of toxic metal uptake^28,32^. Considering the role of NO in the regulation of Si-mediated amelioration of Buta toxicity, it was found that NO fluorescence (Fig. 2C) was greater in Si+Buta combination than the control and Si alone-treated seedlings. However, NO fluorescence (Fig. 2C) was lowered by Buta. This finding indicates that the endogenous level of NO decreased by Buta which coincides with the reduced growth of rice, while Si addition stimulated its level which results in the improved growth of rice seedlings. The observed data thus confirm our hypothesis that NO had a positive role in Si-mediated amelioration of Buta toxicity in rice seedlings.

Although few studies investigated the role of NO in regulating pesticide stress in photosynthetic organisms^58,59^, in this study we report for the first time the interaction of Si and NO and its probable role in the alleviation of Buta toxicity. Induction of Si-mediated NO may be one of the mechanisms reducing Buta toxicity in rice seedlings.

The photochemistry of PSII is a reflection of photosynthetic efficiency. To verify it, the fast chlorophyll *a* fluorescence signals were detected under Buta toxicity (Fig. 1). In the present study, rice seedlings showed decreased values of Phi_P_0_, Phi_E_0_, Psi__0_, ABS/RC, TE_0_/RC, ET_0_/RC and PI_ABS_ under Buta toxicity, while DI_0_/RC showed increment. Decrease in flux ratios, energy flux parameters (except DI_0_/RC) and PI_ABS_ after treatment with Buta indicated alterations in the photochemistry of PS II. These changes resulted into a significant decrease in PI_ABS_. Similarly to our results, Pan *et al*.^60^ reported that an increase in Buta concentration resulted in a decrease in Phi_P_0_, Phi_E_0_, Psi__0_ and PI_ABS_ and an increase in DI_0_/RC. Recently, alterations in chlorophyll *a* fluorescence were also noticed in herbicide-challenged plants^16,17^. However, Si supplementation improved the values of flux ratios, energy fluxes and PI_ABS_ with respect to the control due to improvement in photosynthetic pigments (Table 1), which likely improved the proper function of the photosynthetic machinery, as well as the photochemical reaction center (Fig. 1). Similarly, Tripathi *et al*.^61^ reported that Si addition significantly ameliorated the toxic effects of Cr(VI) on chlorophyll *a* fluorescence parameters. These results suggest Si can protect rice seedlings against Buta toxicity too by protecting PSII photochemistry.

The anatomy of leaf and root shows contrasting characters under different treatments of Si and Buta. Buta toxicity decreased leaf thickness of midrib and blade, the size of the vascular bundle and xylem and phloem cells (Fig. 2 A, B and Tables 2 and 3). Furthermore, under Buta toxicity, a reduction in the size of stomata can lead to more negative impact on transpiration, photosynthesis and gas exchange^62^. Therefore, smaller stomata size would be less efficient in transpiration and gas exchange. Hence, reduction in the photosynthetic pigments (Table 1) along with a reduced size of the stomatal apparatus (Table 2) could have negative impact on PS II photochemistry, which eventually affects biomass accumulation in rice seedlings (Fig. 1). Further, Buta declined the size of root cells such as parenchyma of cortex, xylem and phloem which ultimately resulted in a reduction of the root diameter (Table 3). A marked decrease in cell size might be a result of a decrease in the elasticity of the cell wall as previously demonstrated^63,64^. The reduction in diameter of meta-xylem vessels was also remarkable as it is one of the factors affecting the capacity of transporting nutrients^65^.Therefore, movement of water and minerals from root to shoot through the xylem might be diminished. This is in agreement with the observed decrease in stomatal size under Buta toxicity (Table 2). Limitations in water supply (leading to stomatal closure) and stomatal size might restrict gas exchange and photosynthesis^66^, which led to reduced accumulation of biomass (Table 1). In contrast to this, Si has been reported to maintain the internal structures of plants under different stress conditions^23,33,67,68^. Fleck *et al*.^69^ reported that Si enhanced suberization and lignification in roots of rice. Similarly, Schoelynck *et al*.^70^ also suggested that Si is correlated with lignification in macrophytes, as well as with cellulose content. The results of this study showed that Si supplementation protects leaf and root internal structures under Buta, with the effect being evident in the case of the thickness of leaf midrib and leaf blade, vascular bundles, root diameter, endodermis width and stomatal size (Tables 2 and 3). These anatomical differences result in the formation of barriers to reduce Buta uptake and, ultimately, result in Buta toxicity mitigation.

One of the common responses of plants to stress is the over production of ROS. These ROS act either as signaling or damaging molecules depending on their concentrations inside the cell, regulated by intricate antioxidant defense system^71,72^. Buta significantly increased H_2_O_2_ and O_2_^•−^ contents in rice seedlings, which resulted into a significant increase in MDA content (Figs. 3 and 4). The higher MDA indicates that Buta caused significant cell damage in rice seedlings as indicated by a lower cell viability (Fig. 2D), which coincides with retardation in growth (Table 1). Similarly to this, Wang *et al*.^3^ have also observed higher H_2_O_2_ and MDA level in Italian ryegrass in response to Buta. On the other hand, supplementation of Si with Buta reduced H_2_O_2_ and O_2_^•−^ and MDA which coincided with greater cell viability, indicating that Si protects rice seedlings against oxidative stress under Buta toxicity (Figs. 3 and 2D).

Under normal physiological conditions, the level of ROS is strictly controlled by the antioxidant defense system comprising various enzymes like superoxide dismutase, catalase, peroxidases, etc. and non-enzymatic antioxidants like ascorbate, glutathione, proline, non-protein thiols etc^47,73–75^. The ascorbate-glutathione cycle is of prime importance in regulating H_2_O_2_ level in the cytosol, as well as in the chloroplast under stress and normal conditions^76^. This cycle consists of four enzymes i.e. APX, GR, MDHAR and DHAR and two metabolites (ascorbate and glutathione), which act in a coordinated manner in order to metabolize H_2_O_2_ and decide its fate, i.e. whether it will act as a signaling or damaging molecule. Besides its role in controlling H_2_O_2_ level, this cycle also maintains the redox buffer of the cell by regulating the level of glutathione and ascorbate and their redox ratios, which help in sensing and signaling of stress in order to modulate cellular compartment specific changes by altering the expression of metabolism- and stress-related genes^76–80^. Our results show that Buta stress significantly (*p* < 0.05) inhibited the activities of APX, GR, MDHAR and DHAR and the amount of ascorbate and glutathione which resulted into a great decline in ratios of AsA/DHA and GSH/GSSG (Tables 4 and 5). Reduced activities of the AsA-GSH cycle may be linked with Buta-induced toxicity in rice seedlings due to a reduced availability of the redox buffers like ascorbate and glutathione (Fig. 7) controlling ROS level (Figs. 3A-C and 4). This is supported by the increased lipid damage and lower cell viability detected (Fig. 3 and Fig. 2D). In contrast to the activities, the expression of *OsAPX, OsGR*, and *OsDHAR* was stimulated by Buta (Fig. 6), but that of *OsMDHAR* decreased. These results suggest that, under Buta stress, translation of mRNAs of *OsAPX, OsGR* and *OsDHAR* may be reduced, as indicated by their significant inhibited activities (Table 4). In contrast to our results, stimulation in activities of the ascorbate-glutathione cycle enzymes and transcripts of antioxidants were reported in earlier studies^74,75,79^. However, Si supplementation together with Buta maintains the activities of the ascorbate-glutathione cycle and also stimulates the relative expression of its associated genes (Fig. 6 and Table 4). This results into higher redox couples of ascorbate and glutathione in rice seedlings (Table 5). Under Si supplementation, the increased activities of the ascorbate-glutathione cycle and higher redox couple of ascorbate and glutathione, together with higher NO levels (Fig. 2C), suggest that these modifications regulate of Buta-mediated oxidative stress. Most notably, NO plays the role of signaling molecule in inducing antioxidant defense system, as reported in earlier studies^77,81^.

Proline metabolism has a regulatory function in the homeostasis of oxidation and reduction processes which govern survival of plants under changing environmental conditions^82^. The results show that Buta enhanced the accumulation of free proline and the activity of P5CS, while the addition of Si reversed these effects (Fig. 7). Further, the activity of PDH was lower under Buta toxicity, while in the case of Si+Buta combination it was comparable to the control. These results imply that, under Buta toxicity, the increased accumulation of free proline is needed in order to counterbalance the adverse impact of Buta on rice seedlings, as indicated by the up-regulation in the activity of P5CS and down-regulation of PDH.

Being crucial for enzymes, vitamins, pigments and other biomolecules, mineral nutrients play a vital role in the growth and development of plants and provide biochemical and mechanical strengths by maintaining the structural integrity of the cell^23,33^. Buta treatment decreased the accumulation of mineral elements (Mg, S, P, K, Ca and Fe), except Na (Table 6). However, Si addition maintained the levels of Mg, P, S, K, Ca and Fe, which were higher than their respective control under Buta toxicity (Table 6). These results imply that Si positively regulated nutrient levels under Buta toxicity to maintain the structural and functional integrity of the cell. The increased accumulation of nutrients under Si supplementation is justifiable, as the size of cells and diameter of roots increased under Si treatments and this may help in their absorption (Table 6). Since the Si-mediated up-regulation of nutrients’ uptake is accompanied by a higher level of NO, it can be assumed that NO might be involved in the regulation of their uptake in rice seedlings under Buta toxicity, as reported in earlier studies where NO was found to positively regulate the uptake of certain nutrients^83,84^.

## Conclusions

Herein, we reported for the first time that Si supplementation alleviated Buta toxicity in rice seedlings. Si-mediated alleviation of Buta toxicity was due to adaptive changes in leaf and root anatomical characteristics, up-regulation of Si channel and efflux transporter genes and the ascorbate-glutathione cycle. These changes reduced oxidative stress and impacted nutrient regulation. In Si-mediated amelioration of Buta toxicity, NO has a role in promoting the above-mentioned favorable changes in rice seedlings. These results are valuable from an agronomical point of view, as they will inspire the use of sustainable agricultural practices relying on Si fertilization to sustain crop productivity at herbicide-polluted sites, and thus, this study can open newer avenues for research in this direction.

## Supplementary information

Supplementary Information Table S1.
