# Supplementary material for: Silicon tackles butachlor toxicity in rice seedlings by regulating anatomical characteristics, ascorbate-glutathione cycle, proline metabolism and levels of nutrients
Source: Sci Rep. 2020 Aug 21;10:14078. doi: 10.1038/s41598-020-65124-8 (PMC7442639; doi:10.1038/s41598-020-65124-8)
Supplement: Supplementary file 1 — Supplementary Information Table S1. [file 41598_2020_65124_MOESM1_ESM.pdf]

Title: Silicon tackles butachlor toxicity in rice seedlings by regulating anatomical characteristics, ascorbate-glutathione cycle, proline metabolism and levels of nutrients

Authors: Durgesh Kumar Tripthi[1,7#], Rishi Kumar Varma[2], Swati Singh[3], Manisha Sachan[2], Gea Guerriero[4], Bishwajit Kumar Kushwaha[5], Shruti Bhardwaj[2], Naleeni Ramawat[1], Shivesh Sharma[2*], Vijay Pratap Singh[5#], Sheo Mohan Prasad[6*], Devendra Kumar Chauhan[3*],Nawal Kishore Dubey[7], Shivendra Sahi[8]

Table S1: Primer details and qRT-PCR conditions. The PCR amplification program was as follows:95°C for 10 mins, 40 cycles of 95°C for 30s, annealingfor 30s and 72°c for 30s; followed by melting curve program.Tm- (Melting temperature)

S.N	Gene	Tm	Forward primer (5'→3')	Reverse primer(5'→3')
1	*OsACT11*	55ºC	CAGCCACACTGTCCCCATCTA	AGCAAGGTCGAGACGAAGGA
2	*OsLsi1*	60ºC	CGGTGGATGTGATCGGAACCA	CGTCGAACTTGTTGCTCGCCA
3	*OsLsi2*	62ºC	ATCTGGGACTTCATGGCCC	ACGTTTGATGCGAGGTTGG
4	*OsMDHAR4-1*	60ºC	GTACAACGAACACGGCATTG	TTTCCCCACTGGATGTAAGC
5	*OsDHAR1*	60ºC	CCTTTATCAACGGGCAGAAC	AAATGCTCCAGAGCAACCTG
6	*OsGR1*	62ºC	TCAACATTCCTGGGAAGGAG	CAATATATCCGCCACCAAGG
7	*OsAPX1*	55ºC	AAGGAAGCTATGCCATTCCA	CAAACGTCCAATGTGACACC
